# Dengue Deaths in Puerto Rico: Lessons Learned from the 2007 Epidemic

**DOI:** 10.1371/journal.pntd.0001614

**Published:** 2012-04-17

**Authors:** Kay M. Tomashek, Christopher J. Gregory, Aidsa Rivera Sánchez, Matthew A. Bartek, Enid J. Garcia Rivera, Elizabeth Hunsperger, Jorge L. Muñoz-Jordán, Wellington Sun

**Affiliations:** 1 Dengue Branch, Division of Vector-Borne Diseases (DVBD), National Center for Emerging and Zoonotic Infectious Diseases (NCEZID), Centers for Disease Control and Prevention (CDC), San Juan, Puerto Rico; 2 University of Massachusetts Medical School, Worcester, Massachusetts, United States of America; 3 Puerto Rico Department of Health, San Juan, Puerto Rico; Tropical Medicine Institute Pedro Kourí (IPK), Cuba

## Abstract

**Background:**

The incidence and severity of dengue in Latin America has increased substantially in recent decades and data from Puerto Rico suggests an increase in severe cases. Successful clinical management of severe dengue requires early recognition and supportive care.

**Methods:**

Fatal cases were identified among suspected dengue cases reported to two disease surveillance systems and from death certificates. To be included, fatal cases had to have specimen submitted for dengue diagnostic testing including nucleic acid amplification for dengue virus (DENV) in serum or tissue, immunohistochemical testing of tissue, and immunoassay detection of anti-DENV IgM from serum. Medical records from laboratory-positive dengue fatal case-patients were reviewed to identify possible determinants for death.

**Results:**

Among 10,576 reported dengue cases, 40 suspect fatal cases were identified, of which 11 were laboratory-positive, 14 were laboratory-negative, and 15 laboratory-indeterminate. The median age of laboratory-positive case-patients was 26 years (range 5 months to 78 years), including five children aged <15 years; 7 sought medical care at least once prior to hospital admission, 9 were admitted to hospital and 2 died upon arrival. The nine hospitalized case-patients stayed a mean of 15 hours (range: 3–48 hours) in the emergency department (ED) before inpatient admission. Five of the nine case-patients received intravenous methylprednisolone and four received non-isotonic saline while in shock. Eight case-patients died in the hospital; five had their terminal event on the inpatient ward and six died during a weekend. Dengue was listed on the death certificate in only 5 instances.

**Conclusions:**

During a dengue epidemic in an endemic area, none of the 11 laboratory-positive case-patients who died were managed according to current WHO Guidelines. Management issues identified in this case-series included failure to recognize warning signs for severe dengue and shock, prolonged ED stays, and infrequent patient monitoring.

## Author Summary

Dengue is a major public health problem in the tropics and subtropics; an estimated 50 million cases occur annually and 40 percent of the world's population lives in areas with dengue virus (DENV) transmission. Dengue has a wide range of clinical presentations from an undifferentiated acute febrile illness, classic dengue fever, to severe dengue (i.e., dengue hemorrhagic fever or dengue shock syndrome). About 5% of patients develop severe dengue, which is more common with second or subsequent infections. No vaccines are available to prevent dengue, and there are no specific antiviral treatments for patients with dengue. However, early recognition of shock and intensive supportive therapy can reduce risk of death from ∼10% to less than 1% among severe dengue cases. Reviewing dengue deaths is one means to identify issues in clinical management. These findings can be used to develop healthcare provider education to minimize dengue morbidity and mortality.

## Introduction

Dengue is a major public health problem throughout the tropics and subtropics [1]. During the last decade, both the incidence and severity of dengue in Central and South America, Mexico, and the Caribbean have increased substantially [2]. In Puerto Rico, dengue virus (DENV) was first isolated during a large epidemic in 1963 [3]. Since then, there have been several large island-wide epidemics of dengue with dengue hemorrhagic fever (DHF), including two epidemics in 1998 and 2007 that involved the simultaneous transmission of all four DENV [4], [5]. Despite the well-publicized island-wide epidemic in 2007 and an increasing trend in severe disease [5], the true incidence of fatal dengue is likely under-estimated because of underreporting and under-recognition [6], [7], which has included failure to designate dengue as an underlying cause of death on death certificates [8].

Primary prevention of dengue through vector control activities has had limited success worldwide [9]. Currently, no vaccine exists to prevent dengue nor is there an anti-viral treatment. However, secondary prevention to reduce mortality through improved clinical case management has substantially lowered the mortality rate for severe dengue from 10–20% to <1% in some countries over the past two decades [10], [11]. To begin to understand patient care and management issues related to dengue associated deaths including under-recognition of severe dengue, we performed a review of medical records from the case-series of all laboratory-positive fatal cases in Puerto Rico that occurred during the 2007 epidemic.

## Methods

### Data sources

Suspected deaths due to dengue with onset of illness in 2007 were identified from three sources: 1) the passive dengue surveillance system (PDSS) maintained by the Puerto Rico Department of Health (PRDH) and Centers for Disease Control and Prevention (CDC) Dengue Branch, 2) death certificates filed at the Demographic Registry of Puerto Rico, and 3) hospital-based infection control nurse dengue surveillance system (ICNDSS) as previously described [4], [6]. Complete medical records from all hospitalizations, emergency room and clinic visits for suspected dengue deaths were obtained and reviewed by physician investigators using a standardized instrument to collect demographic, clinical, and laboratory data. This project underwent institutional review at the CDC and it was determined not to be subject to formal institutional review board review requirements as defined by US regulations (i.e., Title 45 Code of Federal Regulations Part 46).

Suspected dengue cases are reported to PDSS by health care providers who submit a serum specimen for diagnostic testing accompanied by a Dengue Case Investigation Report (DCIR). For fatal cases, autopsy tissue is occasionally sent with the serum specimen and DCIR. The DCIR includes patient demographic, clinical, travel, vaccination, and disease outcome data including whether the illness resulted in hospitalization, death or both. DCIR data are entered into an electronic data base. Deaths reported to PDSS are immediately confirmed by calling the reporting hospital.

Hospitalized suspected dengue cases are reported to the ICNDSS by nurse epidemiologists or infection control nurses. Because of a steady decline in participation, data from this reporting system are only used to augment PDSS data. When an ICNDSS case report indicated a patient death these cases were confirmed and investigated.

Death certificates with “dengue” included as a cause or contributing factor in the death were obtained on a monthly basis. The PDSS database was queried to determine if a diagnostic specimen was received for the case.

### Laboratory testing

All serum specimens were tested by DENV serotype-specific, real-time, reverse transcriptase polymerase chain reaction (RT-PCR) [12], [13]. Specimens were also tested for anti-DENV IgM with an IgM antibody-capture enzyme-linked immunosorbent assay (MAC-ELISA) [14]. Specimens with borderline results were retested against a standard negative serum. A quantitative IgG ELISA was performed to detect anti-DENV IgG in all specimens [15]. As human West Nile virus (WNV) infections were identified for the first time in Puerto Rico in early 2007 (i.e., among healthy blood donors), [16] all fatal cases were tested by anti-WNV MAC-ELISA and if positive, WNV specific RT-PCR [17] and plaque reduction neutralization test (PRNT90) assays were performed to differentiate between DENV and WNV infections [18].

All serum specimens with sufficient volume remaining after dengue diagnostic testing was completed were also screened for IgM antibodies to *Leptospira* at the CDC Bacterial Zoonoses Branch, Zoonoses and Select Agent Laboratory using the rapid dipstick ELISA ImmunoDOT kit (GenBio, Inc., San Diego, CA). Specimens with positive or borderline ELISA results were further tested using the microscopic agglutination test (MAT) using 20 *Leptospira* reference antigens representing 17 serogroups [19].

Autopsy tissue was sent to CDC Infectious Diseases Pathology Branch for identification of DENV antigen by immunohistochemical (IHC) microscopy and DENV-specific RT-PCR. If microscopic examination of the tissue or the clinical history was suggestive of leptospirosis (e.g., interstitial nephritis, pulmonary hemorrhage), IHC microscopy was conducted using 16 polyclonal anti-*Leptospira* spp. antibodies. Depending on the clinical presentation and histopathology of the tissue specimens submitted, additional testing with histochemical stains, immunohistochemistry, and/or additional molecular assays was performed to determine the infecting pathogen.

### Definitions

A *suspected dengue case* is a dengue-like, acute febrile illness in a person with that clinical diagnosis and a specimen submitted for dengue diagnostic testing. A *laboratory-positive dengue case* is a suspected dengue case with any of the following: (1) detection of DENV RNA in serum, cerebrospinal fluid, or tissue by RT-PCR; (2) identification of DENV antigen in tissue by IHC assay; (3) IgM anti-DENV seroconversion or demonstration of a fourfold or greater increase in anti-DENV IgG titers in paired serum specimens; or (4) positive anti-DENV IgM in a single serum specimen. A *laboratory-negative dengue case* is a suspected dengue case for which no anti-DENV IgM is detected in a serum specimen collected >5 days after fever onset (i.e., a negative convalescent phase specimen), and no anti-DENV IgM, DENV RNA or DENV antigen is detected from serum collected ≤5 days after onset (i.e. a negative acute phase specimen) or tissue (if submitted). Laboratory-negative dengue cases are hence considered to have the diagnosis of dengue ‘ruled out’. A *laboratory-indeterminate dengue case* is a suspected dengue case that subsequently had no DENV RNA or anti-DENV IgM detected in specimen collected ≤5 days after fever onset, and no convalescent specimen submitted for diagnostic testing.

A laboratory-confirmed leptospirosis case is a suspected dengue case with any of the following laboratory results: 1) isolation of *Leptospira* from a clinical specimen, 2) fourfold or greater increase in MAT titer between acute- and convalescent-phase serum specimens studied at the same laboratory, 3) demonstration of *Leptospira* in tissue by immunohistochemistry, or 4) MAT titer ≥800 in a serum specimen. A *presumptive leptospirosis case* is a suspected dengue case with any of the following laboratory results: 1) presence of MAT titer >200 but <800 in a serum specimen, or 2) demonstration of *Leptospira* in a clinical specimen by darkfield microscopy.

A *terminal event* is defined as the first event in a patient's clinical course that resulted in the need for cardiopulmonary resuscitation (e.g., a hypoxic seizure, an intracranial bleed).

A *hemorrhagic manifestation* is defined by the presence of any of the following: petechiae, purpura, ecchymosis, epistaxis, gingival bleeding, hematuria, menorrhagia, hemoptysis, hematemesis, melena, or an intracranial bleed.

*Dengue fever* (DF) is any suspected dengue case that meets the 1997 World Health Organization (WHO) case definition [20], which include acute onset of fever plus two or more of the following sign or symptoms: headache, retro-orbital pain, myalgia, arthralgia, rash, hemorrhagic manifestation, and leucopenia. Leucopenia was defined by white cell count <5.0×10^9^/L.

*Dengue hemorrhagic fever* (DHF) is any suspected dengue case that meets the following WHO criteria [20]: (1) fever or recent history of fever, (2) any hemorrhagic manifestation, (3) platelet count of ≤100,000/mm^3^, and (4) evidence of increased vascular permeability and plasma leakage which includes: (a) hemoconcentration with a hematocrit ≥20% above the U.S. population mean for age and sex, [21], [22] (b) ≥20% decline in hematocrit following volume-replacement treatment compared to baseline, (c) presence of pleural effusion or ascites detected by any imaging method, or (d) a serum protein or albumin <2.5 percentile for age and sex [23]–[25].

*Dengue shock syndrome* (DSS) is any case that meets the four criteria for DHF and has evidence of circulatory failure manifested by (1) rapid and weak pulse and narrow pulse pressure (≤20 mmHg [2.7 kPa]) or (2) hypotension for age and cold, clammy skin and restlessness.

A *primary DENV infection* (first DENV infection) is a laboratory-positive case in which the anti-DENV IgG titer was <1∶160 in an acute serum specimen collected ≤5 days after the onset of symptoms [14].

A *secondary DENV infection* (≥second DENV infection) is a laboratory-positive case in which the anti-DENV IgG titer was ≥1∶160 in an acute serum specimen [14].

## Results

A total of 10,576 suspected dengue cases were reported with onset of symptoms in 2007; 10,171 to the PDSS only, 68 to the ICNDSS only, and 337 to both systems. There were 40 suspected dengue deaths reported to these surveillance systems and 4 additional deaths were identified only by the Demographic Registry of Puerto Rico. These latter fatal cases had no diagnostic testing performed and are not considered further in this report.

Of the 40 suspected dengue deaths, one case-patient had both tissue and paired serum specimens submitted, eight had tissue and acute serum, two had tissue and convalescent serum, four had tissue alone, one had paired serum specimens, five had only a convalescent serum specimen, and 19 had only an acute serum specimen. Eleven of the 40 suspected deaths were laboratory-positive dengue cases (Table 1); a 0.6% case-fatality rate among the 1,776 hospitalized laboratory-positive dengue cases reported to PDSS. There were no co-infections identified. Of the remaining 29 deaths, 14 were laboratory-negative cases; nine had leptospirosis (three confirmed cases and six presumptive cases) and one had group A *Streptococcus* detected by tissue IHC. Fifteen deaths were laboratory-indeterminate cases although three met WHO clinical criteria for DHF with gastrointestinal hemorrhage and four met criteria for DF with thrombocytopenia and a hemorrhagic manifestation, but had no evidence of plasma leakage documented in the medical records.

**Table 1:** Laboratory Results for the Fatal Laboratory Positive Dengue Cases, 2007, Puerto Rico.

	First Serum Specimen								Second Serum Specimen				Tissue		
Case	DPO	DENV RT-PCR	DENV MAC-ELISA	DENV IgG ELISA	WNV RT-PCR	WNVMAC-ELISA	PRNT	Lepto IgM	DPO	DENV RT-PCR	DENVMAC-ELISA	DENV IgG ELISA	DPO	DENV RT-PCR	DENV IHC
1	3	DENV-3	NEG	NEG	N/A	NEG	N/A	QNS					4	POS	POS
2	5	DENV-2	POS	NEG	N/A	NEG	N/A	QNS							
3	7	NEG	POS	POS	N/A	NEG	N/A	NEG	8†	NEG	NEG	POS			
4	5	DENV-3	NEG	POS	N/A	NEG	N/A	QNS							
5	6	DENV-1	NEG	POS	NEG	POS	D1: 32D2: 128D3: 32D4: 32WNV: 8	QNS							
6	5	DENV-3	POS	POS	N/A	NEG	N/A	QNS							
7	3	NEG	POS	POS	NEG	POS	D1: 64D2: >512D3: 128D4: 16WNV: 16	QNS	4	NEG	POS	POS	4	POS	POS
8	8	NEG	POS	POS	NEG	POS	D1: 256D2: 128D3: 64D4: 64WNV: 16	QNS	9†	NEG	NEG	POS	9	POS	POS
9													4	POS	POS
10	9	NEG	POS	POS	N/A	NEG	N/A	NEG							
11	4	NEG	POS	POS	NEG	POS	D1: >512D2: 256D3: <128D4: 128WNV: <128	QNS							

DPO = day post onset of fever; DENV = dengue virus; RT-PCR = reverse transcriptase polymerase chain reaction; MAC-ELISA = IgM antibody capture enzyme linked immunosorbent assay; ELISA = enzyme linked immunosorbent assay; WNV = West Nile virus; PRNT = plaque reduction neutralization test; IgM Lepto = IgM ELISA ImmunoDOT assay for *Leptospira;* IHC = immunohistochemical microscopy; NEG = negative test result; POS = positive test result; N/A = not applicable for patient; QNS = quantity not sufficient to perform test indicated.

**†:** These samples where whole blood collected at time of death.

### Description of laboratory-positive deaths

Of the 11 laboratory-positive case-patients who died, eight were DENV RT-PCR positive in tissue, serum or both, and three were anti-DENV IgM positive in a single serum specimen (Table 1). Of the five DENV RT-PCR positive in serum, three were DENV-3, one was DENV-2, and one was DENV-1. Among the six case-patients with an acute serum specimen, four had secondary infections and two had primary infections. In the four laboratory-positive case-patients with tissue specimens, DENV was identified by IHC or RT-PCR in lung, liver and kidney specimens, and the most common histopathologic findings were intraalveolar edema and hemorrhage; congestion in the spleen, liver, and/or kidney; and fatty metamorphosis of the liver.

The median age of laboratory-positive case-patients was 26 years (range: 5 months to 78 years). Five were aged <15 years, four were 20–45 years, and two were >70 years. Seven were male. Five of six adults had at least one co-morbidity: two had asthma; one had an autoimmune hypothyroid disease; one had Type 2 diabetes mellitus (DM II) and hypertension; and one had DM II, chronic anemia, congestive heart failure, chronic obstructive pulmonary disease, and hypertension. In addition, four adults were overweight (i.e., body mass index [BMI] of 25.0–29.9), and one adult and one child were obese (i.e., BMI ≥30.0 or a BMI for age >95%).

### Opportunities for early diagnosis and intervention

Seven of the 11 case-patients sought medical care at least once prior to first hospital admission or presenting dead on arrival (DOA) to an emergency department (ED); three were seen by a clinician more than once but only 1 was diagnosed with a “dengue-like-syndrome”. Instead, the most common diagnoses given at these outpatient visits were upper respiratory infection with pharyngitis and/or cough, followed by acute gastroenteritis and viral syndrome. None of the seven case-patients had specimens submitted for dengue diagnostic testing until hospitalization (median 5 days post fever onset; range: 3–9 days) even though they saw clinicians early in the clinical course (median 2 days post fever onset; range 1–5 days). In addition, three case-patients had one or more warning signs for severe dengue at the time of fever defervescence, including persistent vomiting, severe abdominal pain, and narrow pulse pressure, and were sent home. A fourth case-patient, diagnosed with an upper respiratory tract infection as an outpatient, had a seizure at home the day after first being seen as an outpatient and died on the way to the hospital.

Upon final presentation to an ED, the 11 laboratory-positive case-patients had been sick a median of 4 days (range: 1–7 days). Two case-patients presented DOA and four were afebrile, three of which, had signs of shock. A seventh case-patient became afebrile while in ED and developed tachycardia, delayed capillary refill, and a narrow pulse pressure. Six case-patients had warning signs for severe dengue upon arrival to the ED including persistent vomiting (5/9) and abdominal pain (4/9). However, five of the nine case-patients were given a low (least severe) or intermediate severity triage score.

Nine case-patients were admitted to a hospital after a mean ED stay of 15 hours (median 12 hours, range: 3–48 hours) (Table 2). Initial complete blood count done in ED found that six case-patients had platelet counts <100,000/mm^3^ (median 78,000; range 8,000–410,000), five were leukopenic, two were hemoconcentrated (hematocrit 20% above mean for age/sex) and two had a hematocrit <2.5% for age and sex. Six of the nine case-patients met criteria for DF and three met criteria for DHF/DSS. Five case-patients had “dengue” listed in the admission differential diagnosis.

**Table 2:** Clinical Features of the Fatal Laboratory-positive Dengue Cases At Time of First Hospitalization, 2007, Puerto Rico.

	Inpatient Hospitalization										
	At Initial Presentation to Emergency Department (ED)†							During Hospital Admission			
Case	Day post onset	First HCT %	First PLT 10^9^/L	First WBC 10^9^/L	Shock at ED triage	Warning signs at ED triage	LOS in ED	DHF at admit	Freq. VS at admit	No. HCT done	Medical treatment and complications
1	4	55.2	234	12.7	Yes	Yes	12.5	No	Q1	2	Antibiotics, NS and ½NS given while in shock
											Prolonged shock, metabolic acidosis
											Acute respiratory failure and seizure
2	1	35.7	410	10.4	No	No	5.8	No	Q4	2	Antibiotics, steroid, and ½NS given while in shock
											Prolonged shock, metabolic acidosis, DIC
											Blood transfusions and colloid given after seizure
3	7	5.9	28	11.9	DOA	DOA	1.5	N/A	N/A	N/A	Cardiopulmonary resuscitation in ED
4	4	42.5	78	2.5	No	Yes	23.7	No	Q4	4	Steroid and NS given
											Prolonged shock, metabolic acidosis
											Changed VS to q 8 hrs; found without VS in a.m.
											Acute respiratory failure
5	5	29.5	68	1.5	Yes	Yes	6.2	Yes	Q8	4	Steroid, NS and colloid given
											Blood transfusion given for bleed, hypotension
											Prolonged shock, metabolic acidosis
											Encephalopathy, suspected sepsis, antibiotics
											Positive urine culture, acute respiratory failure
6	4	36.5	110	2.9	No	Yes	47.5	No	Q6	11	NS and ½ NS given while in shock
											Drop in HCT thought to be due chronic anemia
											Blood transfusion before discharge
7	4	41.2	102	14.9	No	Yes	13.4	No	Q8	3	Antibiotics, NS and ½NS while in shock
											Prolonged shock, metabolic acidosis
											Fluid overload, acute respiratory failure
											Colloid given after terminal event
8	7	45.4	8	8.3	Yes	Yes	3.0	Yes	Q2	2	Antibiotics and NS given
											Prolonged shock, metabolic acidosis
											Fluid overload, respiratory failure
											No BP taken during night; seizure in am
											Blood transfusions given at final code
9	4	—	—	—	DOA	DOA	0.5	N/A	N/A	N/A	Cardiopulmonary resuscitation in ED
10	5	38.3	61	3.9	No	No	15.0	No	Q8	3	Steroids and NS given
											Urinary retention and muscle weakness
											Neuromuscular work-up not completed
											Acute respiratory failure; died at transfer to ICU
11	3	44.3	49	4.0	No	No	4.3	Yes	Q6	7	Steroids and NS given
											Vaginal and gastrointestinal bleed and then, ICH
											Craniotomy for hematoma evacuation
											Cerebral artery infarction

This table describes the clinical features of laboratory positive dengue case-patients at initial presentation to the Emergency Department (ED) and then whether patient met criteria for DHF and frequency of vital signs upon admission to the inpatient ward. The number of hematocrit levels taken during hospital stay and complications and medical treatment received are listed in last two columns.

**†:** HCT = hematocrit; PLT = platelet; WBC = white blood cell; LOS = length of stay; DHF = dengue hemorrhagic fever; VS = vital signs; NS = normal saline; IVF = intravenous fluids; BP = blood pressure; DIC = disseminated intravascular coagulation; ICU = intensive care unit; ICH = intracranial hemorrhage.

During their hospital stay, several case-patients developed warning signs for severe dengue including persistent vomiting (1/9), abdominal pain (1/9), restlessness (4/9), and mental status changes (4/9). In six cases, warning signs were not recognized as such as there were no new orders or change in the clinical management.

In the end, six of the 11 laboratory-confirmed fatal cases met criteria for DHF or DSS as determined throughout their clinical course and at autopsy (Table 3). Ten of the 11 case-patients had at least 1 hemorrhagic manifestation and nine case-patients had evidence of plasma leakage. Nine of 11 had thrombocytopenia documented.

**Table 3:** Clinical Features of the Fatal Laboratory-positive Dengue Cases at Time of Death or End of Hospital Stay, 2007, Puerto Rico.

	Clinical Features of the Fatal Laboratory-positive Dengue Cases At Time of Death or End of Hospital Stay†											
Case	Met DHF‡ criteria	Lowest PLTs×10^9^/L	Lowest HCT %	Hemorrhagic Manifestations					Signs of Plasma Leakage			LOS triage to terminal event
				GI	Pulmonary	Vaginal	ICH	Other	Effusion	Low albumin	Hemo-concentrated	
1	No	234	46.5	No	No	No	No	Yes	Pleural	Yes	Yes	13.0
2	Yes	53	9.2	Yes	Yes	No	No	Yes	Pleural	Yes	Yes	83.8
3	No	28	5.9	UNK	No	No	UNK	Yes	UNK	UNK	No	DOA
4	Yes	18	20.7	Yes	No	No	No	Yes	Pleural Ascites	Yes	No	59.2
5	Yes	50	29.5	Yes	No	No	No	Yes	No	Yes	No	71.7
6	No	34	24.7	No	No	No	No	No	Ascites	Yes	No	Home
7	Yes	29	33.6	UNK	Yes	No	UNK	Yes	Pleural	Yes	No	30.0
8	Yes	8	45.4	Yes	No	No	UNK	Yes	Ascites	Yes	Yes	19.7
9	No	UNK	UNK	No	Yes	No	No	No	Pleural Ascites Pericardial	UNK	UNK	DOA
10	No	61	34.5	No	No	No	No	Yes	No	No	No	124.8
11	Yes	32	26.1	Yes	No	Yes	Yes	Yes	Pleural	Yes	No	91.8

This table describes the clinical features of laboratory positive dengue case-patients at time of death or end of hospital stay.

**†:** DHF = dengue hemorrhagic fever; PLT = platelet; HCT = hematocrit; GI = gastrointestinal; ICH = intracranial hemorrhage; LOS = length of stay.

**‡:** DHF criteria as defined by World Health Organization in Dengue Hemorrhagic Fever: Diagnosis, Treatment, Prevention and Control. Second edition. Geneva: World Health Organization.

### Monitoring and level of care

Of the nine laboratory-positive case-patients admitted to a hospital, only three had capillary refill time assessed in the ED or at admission. Vital signs were measured at a median of every 3 hours in the ED but most case-patients had less frequent measurements after admission (Table 2). Two case-patients were admitted from the ED to the ICU and had vital signs measured every 1 or 2 hours.

Eight of nine hospitalized case-patients died during hospitalization, and the other case-patient was found dead at home within 18 hours of hospital discharge (Table 3). Three of the eight case-patients who died in the hospital had no recorded blood pressure measurements during the 8 hours before their terminal event. Five case-patients had their terminal event on the inpatient ward and another case-patient was transferred to ICU and had a terminal event within minutes of the transfer. Of those who died in hospital, six of eight case-patients died during a weekend (between 1701 on Friday and 0759 Monday), and five had a terminal event between 2300 and 0759.

### Interventions and supportive care

All nine hospitalized case-patients received intravenous fluids, most commonly 0.9% normal saline (Table 2). Four received 0.45% normal saline while in shock. Four received intravenous albumin (5% or 25% solution) during their hospitalization but only one received colloids prior to the terminal event. Three of the nine hospitalized case-patients had signs of fluid overload prior to death including periorbital edema, dyspnea, and abdominal distention documented in their medical record.

There was frequent use of corticosteroids in laboratory-positive case-patients (Table 2). Five of the nine hospitalized case-patients received intravenous methylprednisolone as inpatients and one received dexamethasone in the ED before hospital admission. One case-patient who was DOA received oral prednisone during an outpatient visit.

Hematocrit levels were assessed every 17 hours on average (range 10–42 hours) for the six hospitalized case-patients with clinically significant hemorrhage (Table 2 and 3). Three of these six case-patients were given packed red blood cells; two in response to frank blood per nasogastric tube or rectum and one during the final code. One of these case-patients also received fresh frozen plasma in response to clinically significant bleeding. No case-patients were given whole blood. Two of the case-patients who received packed red blood cells also received platelets; one in response to clinically significant bleeding and one during the final code. Four additional case-patients had platelets ordered but they did not receive them prior to death.

### Complications and reported causes of death

Many of the nine hospitalized case-patients developed complications including metabolic acidosis (6/9), prolonged shock (6/9), acute respiratory failure (6/9), fluid overload (3/9), and seizures (3/9) (Table 2). Only 1 of the 11 case-patients had evidence of a secondary bacterial infection even though most patients had blood and urine cultures taken. Dengue, DHF, DSS, or status-post dengue syndrome was listed as the cause of death or a contributing factor in only five case-patients. The remaining six death certificates listed causes or contributing factors including hypovolemic shock, hypotension, metabolic acidosis, septicemia unspecified, bronchopneumonia unspecified, viral infection unspecified, brain death, and ischemic cerebral infarct.

## Discussion

This case review showed that although there was a relatively low case-fatality rate among hospitalized patients with dengue during the 2007 epidemic in Puerto Rico, the clinical management of all fatal dengue cases deviated from the current WHO guidelines. Although chronic disease and bacteremia have been associated with poor outcomes, including death among adults with severe dengue [26]–[30], only two adult case-patients had co-morbidities that may have contributed to their deaths. In short, the majority of laboratory-positive fatalities appeared to be due to dengue, and none of our case-patients were managed according to the 1997 WHO Guidelines.

This review illustrates various levels of delay in receipt of appropriate level of care. Delay in receipt of the appropriate level of care and prolonged shock has been associated with poor outcomes among patients with severe dengue [31]–[33]. Most of our case-patients were seen by a clinician at least once before being hospitalized or presenting DOA, and only one case-patient was identified as having dengue. In fact, four of the seven case-patients seen as outpatients could have benefited from timelier referral to an inpatient facility including the three that sought care multiple times but were sent home even though they had warning signs of severe dengue and another that presented DOA the day after being seen. These findings indicate the need for educating patients and clinicians in identifying dengue and recognizing warning signs for severe dengue so that anticipatory guidance can be given to minimize delay and appropriate care can be initiated in a timely manner.

Poor disease recognition and failure to detect increased disease severity in the ED appeared to contribute to the delay in receipt of appropriate inpatient care. Contributing factors included patients being given low triage scores and infrequent monitoring of vital signs. Lack of inpatient beds was only documented in two cases and probably did not contribute to poor outcomes. We are not aware of any published studies that indicate that use of triage scores/systems may be a factor in treatment delay for dengue as has been shown for severe sepsis [34]. Automated triage systems detect patients with high body temperature and low systolic blood pressure; whereas, it would be useful in dengue endemic countries for these systems to also identify patients with hypothermia, narrow pulse pressure and age-specific tachycardia in the absence of hyperthermia. In countries with a sizeable number of adult patients with severe dengue, it would be important for triage systems to identify patients with chronic hypertension as low- normal systolic blood pressure may be abnormal for these patients. The early markers for severe disease and mortality among dengue patients needs to be better defined [28], [35] and used to develop rules for triage of patients in dengue endemic areas, especially during epidemics.

Monitoring patients closely for warning signs of severe disease and early signs of shock until at least 24 hours after fever defervescence is important as patients may rapidly deteriorate at this time. Most of our case-patients were admitted to the general inpatient ward, had infrequent monitoring of vital signs, and hematocrit levels were not ordered at a frequency necessary to monitor plasma leakage with its attendant hemoconcentration and response to fluid resuscitation. In addition, we found that warning signs and early signs of shock were not acted upon in a timely manner even when clearly documented by the nursing staff. Many hospital deaths occurred during the night or weekend, and a few had no blood pressure measurement recorded for several hours before death.

For patients with severe dengue, the mainstay of successful management is early and judicious replacement of plasma leakage with isotonic crystalloid solutions, including normal saline, Ringer's lactate, Ringer's acetate, or 5% glucose in normal saline, followed by colloid solutions in the event that shock is refractory [36]–[38]. Our review found that some case-patients were given intravenous non-isotonic crystalloid solutions during shock, and only one patient was given intravenous colloid solution before the terminal event even though six of the eight case-patients who died in hospital had refractory shock.

Most case-patients received corticosteroids even though their use has been shown to be no more effective than placebo or no treatment in reducing the number of deaths and the need for blood transfusion [39]. Two recent reviews found insufficient evidence to justify the use of corticosteroids in managing dengue shock syndrome and recommended that corticosteroids not be used to treat dengue [39], [40]. In addition, there is no convincing physiological rationale for their use and there are multiple potential side effects including stress ulceration and upper gastrointestinal bleeding in critically ill patients, hyperglycemia, and immunosuppression with an increased risk for infection.

Although most case-patients had clinically significant bleeding, few received packed red blood cells and none received whole blood. Infrequent monitoring of hematocrit and vital signs may have contributed to the late detection of hemorrhage. Instead, complete blood counts may have been ordered to monitor platelet counts. Platelet transfusions were ordered for most of the hospitalized case-patients but many died before receiving them. Guidelines generally recommend platelet transfusions be given to patients who have clinically significant bleeding; however, use of prophylactic platelet transfusions for dengue is a subject of debate [41]. Recent studies have found that in children, platelet counts are not predictive of bleeding [32], [42], [43] nor do they correlate with bleeding severity [44]. Instead, prolonged shock was found to be a risk factor for severe hemorrhage [32]. In a study of 106 pediatric patients with DSS and coagulopathy, prophylactic platelet transfusions for platelet counts <30×10^9^ L^−1^ and fresh frozen plasma did not prevent hemorrhage and may have contributed to the development of pulmonary edema resulting in increased hospital stays [45]. Moreover, prophylactic platelet transfusions do not seem to expedite platelet recovery [45], [46]. Studies among adults have conflicting results; some found no association between a platelet count and bleeding [46], while others found an association between platelet counts of 50×10^9^ L^−1^ or less than 20×10^9^ L^−1^ and bleeding [42], [47].

Lastly, our review illustrates the difficulties in making a laboratory diagnosis of dengue, especially late in the course of the disease. Even though we tested acute and convalescent specimens by all available diagnostic tests, 37.5% (15) had an indeterminate diagnosis. This occurred because the available specimen(s) were incorrectly collected with respect to the course of dengue. Seven of the 15 case-patients with an indeterminate dengue diagnosis had specimens obtained at day 4 or 5 post onset of symptoms, a period when viremia or anti-DENV IgM may be undetectable. Physicians practicing in dengue endemic areas need to be aware of including dengue in their differential diagnosis of acute febrile illness and that they must obtain serum samples for diagnostic testing early (days 1 to 3) after symptom onset. To improve diagnostic accuracy for patients who present late, samples should be collected immediately and a second collected 5 to 7 days later. Whether more sensitive diagnostic assays can be developed to increase diagnostic accuracy for specimens collected only during the critical period of dengue remains to be determined.

There are limitations to our study. This was a small case-series study which only generates descriptive data and does not identify risk factors associated with dengue deaths. The identification of risk factors must await case-control studies in a larger number of dengue fatalities. However, the high frequency of certain findings (e.g., use of non-isotonic fluids for refractory shock, infrequent monitoring vital signs, not identifying warning signs for severe dengue) suggests that they may be risk factors for poor outcomes. The other limitation is that our catchment systems may not have detected all fatal dengue cases, which may have introduced biases due to incomplete case ascertainment. However, our present surveillance approach again confirmed that in Puerto Rico, dengue continues to not be listed as the cause or underlying cause of death among laboratory-confirmed fatal cases [4], [8]. Better estimates of the degree of under-recognition and reporting of dengue deaths must await additional assessment of reporting studies. However, the consistency of issues in clinical management in our case-series suggests that biases due to underreporting are probably minimal.

Clearly there is need to improve surveillance of severe and fatal cases, and to evaluate clinicians' diagnosis and clinical management of dengue in Puerto Rico. Our presumption is that, even in a dengue endemic area where the disease should be well known, sustained health care provider education and training are necessary to improve detection, diagnosis, and management of dengue and lower dengue morbidity and mortality. CDC Dengue Branch and PRDH in collaboration with a number of medical organizations used findings from this fatal case review and a physician survey that we conducted in 2007–08 (CDC data, not published) to develop a post graduate course on the clinical management of dengue for physicians in Puerto Rico which was implemented in 2009–10.

## Supporting Information

Checklist S1 **STROBE checklist.**
(DOC)
